# An Investigation into the Optical Identification of Flaws in Excavated Ceramic Artifacts via Limited-Data Simulation

**DOI:** 10.3390/s25165172

**Published:** 2025-08-20

**Authors:** Haotian Yuan, Xiaohan Dou, Gengpei Zhang, Yuanyuan Zhang

**Affiliations:** School of Electronic Information and Electrical Engineering, Yangtze University, Jingzhou 434100, China; 13212783009@163.com (H.Y.); 13227676170@163.com (X.D.); judgebill@126.com (G.Z.)

**Keywords:** machine learning, Infectious disease prediction, deep learning

## Abstract

The Terracotta Army, an integral part of China’s cultural heritage, has suffered physical erosion like cracks and notches over time. Manual inspection methods are inefficient and subjective. This study proposes an automated defect detection system based on computer vision to enhance the efficiency and precision of detecting these defects. The system includes the following core modules: (1) high-resolution image acquisition, which ensures comprehensive and detailed data capture; (2) sophisticated image illumination processing, which compensates for varying lighting conditions and improves image quality; (3) advanced image data augmentation techniques, which enrich the dataset and improve the generalization ability of the detection model; and (4) accurate defect detection, which leverages state-of-the-art algorithms. In the experimental phase, the efficacy of the proposed approach was evaluated. Illumination-enhanced low-light images were used for data augmentation, and the generated images showed high similarity to the original images, as measured by PSNR and SSIM. The YOLOv10 algorithm was employed for defect detection and achieved average detection rates of 91.71% for cracks and 93.04% for abrasions. This research provides a scientific and efficient solution for cultural relic protection and offers a valuable reference for future research in heritage conservation.

## 1. Introduction

The Terracotta Army, an essential part of the world’s cultural heritage, boasts a long historical origin and unique cultural significance. As the crystallization of the exquisite craftsmanship of ancient artisans [1], it not only reflects the social outlook and artistic style of that era but also showcases the highly advanced techniques in ancient craftsmanship. These cultural relics carry rich historical, artistic, and technological values and serve as crucial materials for studying the development of ancient society, culture, trade [2], and technology. They also mirror the social structure [3] and cultural characteristics of the time. Through the analysis of ceramic cultural relics [4], archaeologists can gain in-depth insights into the exchanges and interactions of ancient cultures, as well as the living habits and beliefs of ancient humans.

However, the preservation of pottery poses a formidable challenge. Due to the fragility of its materials and the complexity of its craftsmanship, these cultural relics are vulnerable to damage from factors such as temperature and humidity fluctuations, environmental pollution, improper handling, and long–term storage [5]. Such damage manifests as cracks, notches, and other defects. These impairments not only threaten the physical structural integrity of the cultural relics but also affect their aesthetic and research values. Especially in extreme environments, such as rapid climate changes or unexpected mechanical impacts, the preservation status of cultural relics becomes even more precarious.

**Figure 1:**
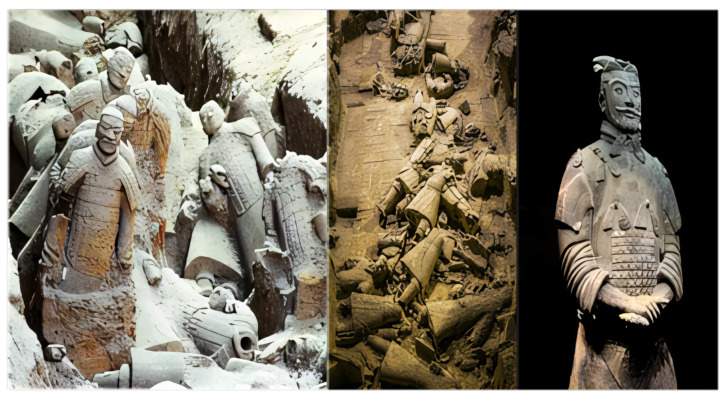
Unearthed image of Terracotta Warriors.

This is a set of pictures showing the Terracotta Army of Emperor Qin Shi Huang (as shown in Figure 1). The Terracotta Army is an important cultural relic from the Qin Dynasty (221 BC–207 BC) in China and is located at the site of Emperor Qin Shi Huang’s Mausoleum in Lintong District, Xi’an City, Shaanxi Province. The first and second pictures illustrate the damaged and fragmented state of the Terracotta Army upon excavation, while the third picture shows the restored Terra.

Currently, the efficiency of manual inspection in cultural heritage protection is relatively low. Wang et al. [6] pointed out that traditional defect detection mainly relies on expert experience and naked-eye observation. Although this method has been foundational in early cultural relic protection efforts, it suffers from low efficiency, subjectivity, and lack of consistency—especially in detecting micro-cracks and internal defects where accuracy is severely limited. To address this, modern non-destructive testing (NDT) technologies have been increasingly applied. Feng et al. [7] emphasized that NDT offers more precise and scientific assessment methods, playing a key role in preserving the integrity of artifacts.

In broader industrial applications, defect detection has been widely used in fields such as road maintenance, sanitary ceramics, metal inspection, and civil infrastructure. Ren et al. [8] proposed a GAN-based data augmentation method to alleviate small and non-i.i.d. data problems. Guo et al. [9] introduced SPEED, a dilated convolutional network for real-time metal surface defect detection. In civil structures, Guo et al. [10] and Jiang [11] emphasized the importance of data quantity and proposed models like JAFFNet to address scale variation and background complexity. Liu et al. [12] developed AIS-Net for pixel-level segmentation of fine defects. Zhao et al. [13] discussed incremental learning for unknown defect discovery, while Zhao et al. [14] collected high-resolution datasets via unmanned platforms for infrastructure inspection. In addition, Wang et al. [15] and Liu et al. [16] improved YOLO models (BL-YOLOv8 and YOLO-pdd) to optimize detection speed and accuracy. These improvements underscore the efficiency and adaptability of YOLO-based models, making them well-suited for complex defect detection tasks.

However, the application of deep learning in pottery cultural relics faces unique challenges. Due to the rarity and fragility of artifacts, access to data is highly restricted. This leads to limited image availability and high annotation cost, making it extremely difficult to construct large, labeled datasets for defect detection.

To overcome these limitations, image data augmentation has become a vital strategy. In particular, diffusion models have shown exceptional performance in addressing data scarcity and enhancing model generalization. Razzhigaev et al. [17] proposed Kandinsky, integrating image priors with latent diffusion for multi-modal generation. BLIP-Diffusion [18] improved multi-modal control and fine-tuning efficiency. Unicontrol [19] introduced a unified diffusion framework adaptable to diverse tasks. RAPHAEL [20] and OFT [21] enabled precise text-to-image generation and controllability, respectively. Blattmann et al. [22] extended these principles to video generation via Stable Video Diffusion. Xu et al. [23] introduced DMV3D for text-guided 3D image synthesis, while Liu et al. [24] developed HyperHuman using a large-scale human dataset. In medical imaging, Öttl et al. [25] demonstrated diffusion-based segmentation with uncertainty modeling, further supporting their generalization potential.

In addition to generation methods, data acquisition techniques such as 3D scanning and image-based NDT have also advanced cultural relic research. Wang et al. [26] and Wang and Guo [27] demonstrated that 3D scanning provides high-precision models essential for digital restoration. Remondino [28] reviewed its applications globally, affirming its value for preservation. For non-invasive pigment analysis, Li et al. [29] and Navarrón et al. [30] used hyperspectral imaging on ancient murals. El-Hakim and Remondino [31] further demonstrated the utility of terrestrial laser scanning for artifact digitization.

While deep learning techniques, particularly CNNs, have been widely applied to ceramic identification and surface defect detection, their full potential is constrained by limited data availability in the heritage domain. Therefore, leveraging image generation and augmentation techniques, especially diffusion-based models, emerges as a promising solution.

The main contributions of this paper lie in the proposition of an automated defect detection system grounded in computer vision. This system is designed to enhance the efficiency and accuracy of detecting flaws in unearthed ceramic artifacts. By amalgamating advanced image acquisition techniques, image illumination processing, data augmentation methods, and the YOLOv10 detection algorithm, this study addresses the limitations of traditional manual inspection methods, which are often inefficient and subjective. Furthermore, it offers a scientific and effective solution for the preservation of cultural heritage. Specifically, the following:

1. Innovative Image Data Augmentation: By integrating FastGAN technology, this study effectively overcame the challenge of limited artifact image data, significantly enhancing the generalization ability of the model and the diversity of the dataset. This ensures stable and high-quality image reconstruction even with limited samples.
2. Efficient Defect Detection Algorithm: The employment of the YOLOv10 algorithm enabled precise identification of surface cracks and abrasions on ceramics, achieving detection accuracies of 91.71% and 93.04%, respectively. This outperforms traditional methods and other baseline models.
3. Cross-Material Applicability: Beyond ceramics, the proposed method demonstrates adaptability to cultural relics made of other materials like stone and bronze. This establishes a versatile and efficient defect detection framework for the field of cultural heritage preservation.

These contributions not only advance the technology of cultural heritage preservation but also offer important references for future research in this domain.

## 2. System’s Overall Approach

The work of this paper mainly consists of two major parts: hardware and software. The aim is to develop an efficient defect detection system for pottery jars. In terms of hardware, the Raspberry Pi is used as the core of the lower-level machine in the system. It controls a high-resolution camera to conduct comprehensive image acquisition, ensuring high-quality data input. The software part includes image pre-processing, image data augmentation, and defect detection modules. The image pre-processing is used to remove noise and enhance the image quality. The data augmentation is employed to expand the training dataset and improve the model’s generalization ability. The defect detection module utilizes deep learning algorithms to accurately identify defects in the pottery jar images. The design of the entire system is dedicated to achieving efficient and accurate defect detection of pottery jars.

### 2.1. Defect Image Acquisition

**Figure 2:**
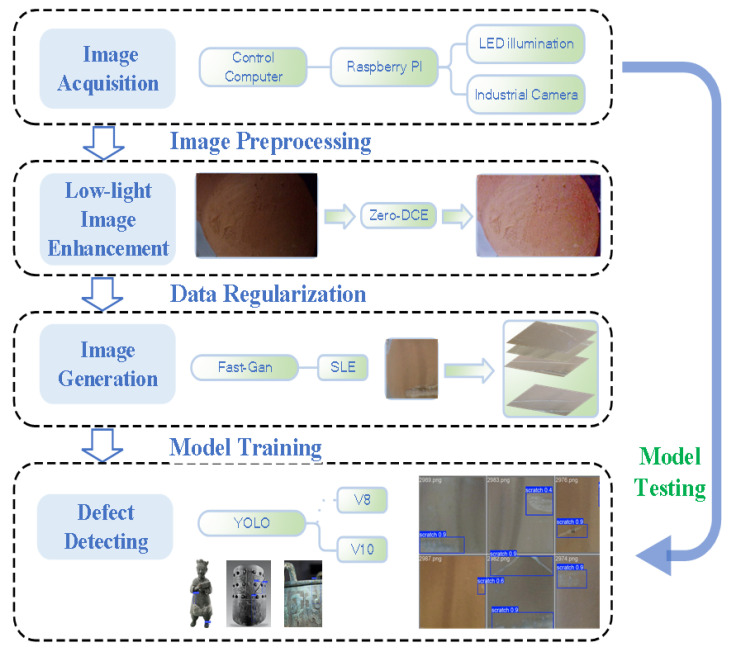
Visual inspection framework for unearthed pottery defects based on few-shot modeling.

This paper introduces a pottery jar image acquisition system based on an industrial camera and a Raspberry Pi. An industrial camera image acquisition system based on Raspberry Pi 4B is constructed. In this paper, the Basler acA2500-60gc industrial camera, which features high resolution and good low-light performance, is selected. This camera is equipped with a 25-megapixel CMOS sensor, capable of providing high-definition images and suitable for capturing the details of pottery jars. The Basler acA2500-60gc camera supports full-resolution image capture at 60 frames per second, making it highly suitable for applications that require rapid image acquisition. When collecting ceramic images, the system receives commands issued from the Terminal. These commands are transmitted to the Slave Computer, which serves as the core control unit. The Slave Computer receives and processes the commands from the Terminal and then controls the Industrial Camera and the Illuminating System, respectively. The Industrial Camera is responsible for image acquisition, while the Illuminating System provides appropriate light source support for the camera. In this way, the coordinated operation of the devices is achieved, and the precise image acquisition task is completed. The specific design principle is shown in Figure 2.

### 2.2. Image Illumination Enhancement

**Figure 3:**
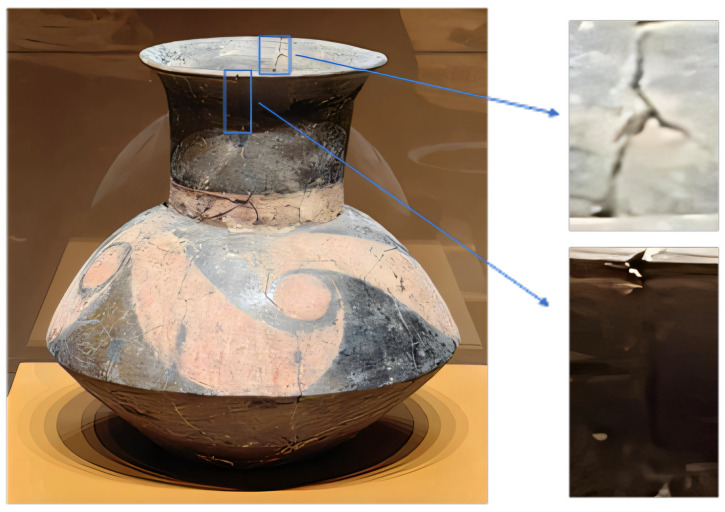
Detailed diagram of defects in unearthed pottery.

In ceramic defect detection based on computer vision, illumination enhancement is a pivotal step in improving detection performance, particularly under low-light conditions. As depicted in Figure 3, the pottery unearthed from Taosi, especially the painted pottery plates, not only possess unique artistic value but also offer invaluable insights into the ancient cultures of the Central Plains region through their decorative patterns and manufacturing techniques. These pottery pieces are crafted from fine-textured clay and adorned with painted decorations. Common embellishments include black pottery coatings and colorful patterns in shades of red, white, and yellow. The motifs range from dots, stripes, and geometric patterns to vortex, cloud, meander, dragon, and variant animal patterns. These patterns share resemblances with the designs on bronze artifacts from the Shang and Zhou dynasties, mirroring the cultural continuity and evolution of that era. Nonetheless, when conducting defect detection on these pottery items under low-light circumstances, the clarity and contrast of the images play a decisive role in the accuracy of the detection results. A low-light environment can lead to a reduction in image contrast, blurring the distinction between the intricate painted patterns on the pottery surface and the background. This, in turn, hampers the identification and restoration of cultural relic defects.

Illumination enhancement can significantly improve the contrast of images, making defect features more prominent and facilitating subsequent identification and localization. Additionally, low light conditions can cause image details to blur and edge information to be lost. Edge information, however, is a crucial clue in defect detection. The enhanced images can better preserve edge information, enhance the effectiveness of edge detection, and reduce the occurrence of false positives and false negatives. Secondly, illumination enhancement can improve the brightness and color representation of images, making them brighter, clearer, and the colors more vivid and natural. This not only enhances the visual experience of viewers but also provides more accurate color information for defect detection, helping to more realistically reflect the state of the ceramic surface. Moreover, high-quality images simplify the image pre-processing steps, reduce the complexity of algorithms and the consumption of computing resources, thereby improving the detection speed and efficiency. In practical applications, lighting conditions may vary. Illumination enhancement enables the defect detection system to better adapt to different lighting environments, improving the system’s robustness and stability and expanding its application scenarios. In conclusion, illumination enhancement is of great necessity in ceramic defect detection. By improving image quality, it provides strong support for enhancing the accuracy, speed, and efficiency of detection.

Many methods have been proposed to address the challenge of improving image quality under low-light conditions. Wang et al. [32] proposed a Deep Lighting Network (DLN) to enhance low-light images by estimating the residual between low-light images and normal-illumination images through a Convolutional Neural Network (CNN). Similarly, Zhu et al. [33] proposed an Edge-Enhanced Multi-Exposure Fusion Network (EEMEFN) to enhance extremely low-light images by improving image brightness and revealing hidden information in dark areas. Xu et al. [34] proposed a frequency-based decomposition enhancement model, focusing on restoring image objects in the low-frequency layer and enhancing high-frequency details.

Traditional low-light image enhancement methods usually rely on a large number of paired datasets for training. However, the Zero-DCE [35] method adopts an innovative approach. By leveraging the context information of the dark areas in images, it designs a zero-shot learning framework that can effectively enhance low-light images without the need for paired data. The core idea of Zero-DCE is to extract the context information of the dark areas from low-light images through a Deep Convolutional Neural Network (CNN) and generate corresponding enhanced images. This method proposes a new loss function that combines the adaptive adjustment of image content and the extraction of dark-area information, enabling the effective restoration of details and brightness in low-light images. In this paper, the Zero-DCE method can achieve good results on the ceramic dataset in an unsupervised manner.

### 2.3. Image Data Augmentation

The diversity and stylistic variation of Chinese excavated artifacts—particularly ceramic relics—pose unique challenges for generative modeling. These artifacts span a wide range of dynasties, each characterized by distinct aesthetic conventions, forms, and decorative techniques. Moreover, archaeological excavations are often time-constrained and dataset availability is uneven across different periods, necessitating the rapid construction of style-specific generative systems with limited training data.

Table 1 presents a comparative analysis of FastGAN, StyleGAN2, and diffusion models, evaluating their performance across key criteria relevant to the demands of archaeological and cultural heritage applications.

Given this context, FastGAN emerges as the most suitable choice for artifact image synthesis. Unlike more complex models such as StyleGAN2 or diffusion-based architectures (e.g., Stable Diffusion), FastGAN is capable of high-quality generation with minimal data, significantly reduced training time, and lower computational requirements. This makes it ideal for rapid prototyping and deployment in cultural heritage research, especially when modeling artifacts from underrepresented dynasties or less-documented historical phases.

#### 2.3.1. The Deep Network of the Generator and the Gradient Flow Problem

**Figure 4:**
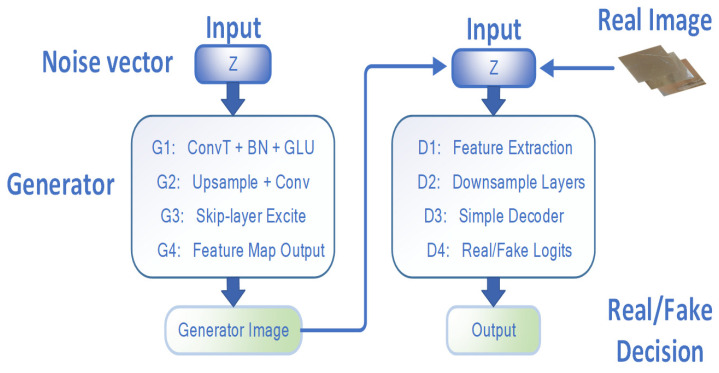
A holistic framework for data augmentation.

When generating high-resolution images of pottery surface defects, the deep network of the generator has a relatively large number of layers. This leads to poor gradient flow during the training process, which affects the training speed and stability. To address this issue, this paper adopts the kip-Layer Channel-wise Excitation (SLE) module. The overall ceramic image generation process is shown in Figure 4.

#### 2.3.2. The Role and Structure of the SLE Module

To enhance the training stability and generation performance of the deep generative network, this paper introduces the Skip-Layer Excitation (SLE) mechanism. This mechanism is designed to achieve efficient information flow across different resolutions. Through the SLE mechanism, the network can effectively transfer information between different resolution levels, thus significantly strengthening the stability of the generation process and improving the generation quality.

The SLE module extracts the global information of the low-resolution feature maps through a cross-resolution channel attention mechanism and performs channel-wise weighted modulation on the high-resolution feature maps to enhance the expression of key information. Specifically, the low-resolution feature maps first undergo adaptive pooling. Subsequently, a series of convolutional operations (4 × 4 and 1 × 1) and a Sigmoid activation are applied to generate a channel attention weight map, which is then used to perform channel-wise weighting on the high-resolution feature maps.

**Figure 5:**
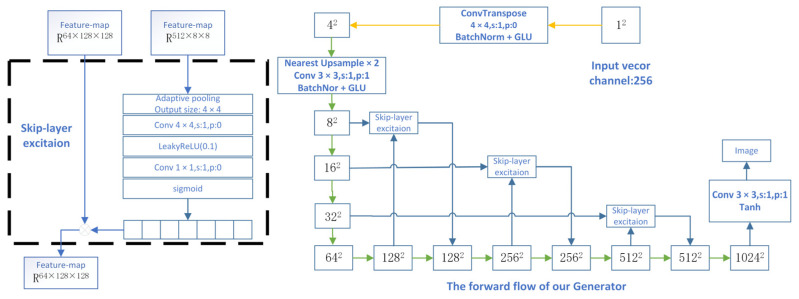
Implementation of the SLE module and the network structure of the generator.

The SLE module is ingeniously embedded in the middle-resolution layers (8^2^, 16^2^, 32^2^, 64^2^) of the generator. This effectively alleviates the problem of gradient vanishing in the deep network and refines the feature expression. As shown in Figure 5, the SLE module extracts the global information of the low-resolution feature maps through the cross-resolution channel attention mechanism and performs channel-wise weighted modulation on the high-resolution feature maps. The specific implementation process and the network structure of the generator are as depicted.

Meanwhile, define the Skip-Layer Excitation module.

(1) $$b=F(a_{low},\{W_{i}\})\cdot a_{high}$$

Here, a and b represent the input and output feature maps of the SLE module, respectively. The function F operates on $a_{low}$, and $W_{i}$ represents the module weights to be learned. The left panel of Figure 3 shows the implementation of the SLE module in practical applications, where $a_{low}$ and $a_{high}$ correspond to the feature maps with resolutions of 8 × 8 and 128 × 128, respectively.

In F, an adaptive average pooling layer is first applied to downsample $a_{low}$ to a 4 × 4 resolution. Subsequently, it is further downsampled to a 1 × 1 resolution via a convolutional layer. To introduce non-linearity, the LeakyReLU activation function is utilized. After that, another convolutional layer maps $a_{low}$ to a tensor with the same number of channels as $a_{high}$. Finally, through the gating operation of the Sigmoid function, the output of this process is multiplied by $a_{high}$ along the channel dimension, generating an output with the same shape as b.

#### 2.3.3. Generator Structure and Forward Propagation Process

In the forward propagation process of the generator, the input vector starts with an initial dimension of 12. Through successive nearest neighbor interpolation, upsampling and convolutional operations (with a 3 × 3 convolution kernel and the GLU activation function), the resolution of the feature map is gradually increased until a high-resolution image of 10242 is generated. In the last layer, a 3 × 3 convolution and the Tanh activation function are used to map the feature map into the final image output.

Compared with the traditional GAN architecture, the SLE module significantly reduces redundant operations in the network. Meanwhile, it enhances the coordination between global information and local textures, improving the quality and consistency of the generated images. This generator structure has the dual advantages of lightweight computation and high-resolution generation. It is particularly suitable for high-resolution image generation tasks that require fine textures and consistency in global style, such as fine-grained image synthesis and efficient image generation in resource-constrained environments.

#### 2.3.4. Streamlined Self-Supervised Discriminator

This paper proposes a method of applying streamlined and strong regularization to [discriminator name]. We regard [discriminator name] as an encoder and use a small decoder to train it. This auto-encoding training method prompts [discriminator name] to extract image features, while the decoder can effectively reconstruct the image. The decoder is co-optimized with [discriminator name] and is trained through a simple reconstruction loss, and the training is conducted only on real samples. The specific formula is as follows:

(2) $$L_{recons}=E_{f\sim D_{encoder}(x),x\sim I_{real}}[\left|\left|G(f)-T(x)\right|\right|]$$

Among them, f is the intermediate feature map obtained from D. The function G encompasses the processing of f and the decoder, while the function T represents the processing of the sample from the real image.

**Figure 6:**
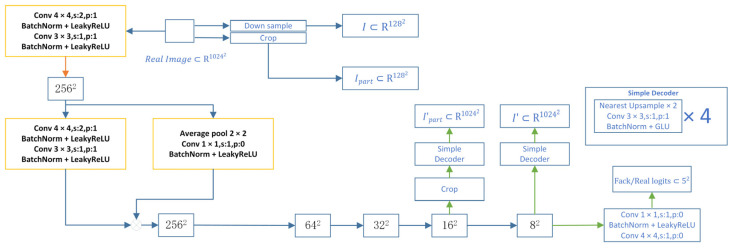
Architecture diagram of FastGAN feature extraction and generation network.

As shown in Figure 6, this architecture aims to determine the authenticity of the input image and repair some damaged images. The input image is downsampled through multiple convolutional blocks. Each convolutional block contains a 4 × 4 convolution kernel with a stride of 2, and LeakyReLU activation and Batch Normalization are used to stabilize the training. The downsampling process gradually reduces the image resolution from 1024 × 1024 to 8 × 8.

At the 128 × 128 resolution stage, feature maps at different levels are fused through a feature concatenation operation to further enhance the ability to extract multi-scale information. Finally, the discriminator performs two convolutional operations at the 8 × 8 resolution and outputs real/fake logits for the binary classification task.

In addition, a simple decoder is introduced in the architecture for local image restoration. Through downsampling and cropping operations, local regions are extracted from real images and fed into the decoder. The decoder uses nearest neighbor upsampling and convolutional operations to gradually restore image details. Starting from 8 × 8, the decoder goes through four upsampling steps to recover to 128 × 128. The reconstructed image output by the decoder is compared with the input local region to ensure the consistency of local features and details.

### 2.4. Defect Detection

In the field of defect detection, considering the stability and update speed of the YOLO algorithm, YOLO is mainly adopted as the primary algorithm for defect detection.

YOLOv8 [36,37] inherits the backbone network and Neck (feature pyramid) design of YOLOv5. It uses CSPNet (Cross Stage Partial Network) as the backbone network, which improves the network’s computational efficiency and feature reuse ability. The ELAN module (Efficient Layer Aggregation Network) is introduced to further optimize multi-scale feature extraction. For feature fusion, PANet (Path Aggregation Network) is adopted to facilitate the interaction of upstream and downstream feature information. The output part mainly consists of three detection heads at different scales, which are used to handle targets of different sizes. YOLOv8 pays more attention to lightweight design and speed optimization, achieving a balance between detection speed and accuracy.

YOLOv10 [38,39,40] is optimized based on YOLOv8, with a focus on enhancing the feature extraction ability and multi-scale feature fusion. The EVC module (Explicit Visual Center) is introduced in the backbone network, which further strengthens the feature representation ability, especially being more effective in extracting detailed features. The feature pyramid network (Neck) is further enhanced. By introducing a module similar to the LVC module (Lightweight Visual Center), the detection performance is improved under the lightweight design. The design of YOLOv10 pays more attention to high-resolution tasks and the processing of detailed information in object detection, making it particularly suitable for small-target and high-resolution scenarios. Meanwhile, more effective adjustments are made to the upstream and downstream paths, enhancing the interaction between the Top–Down and Bottom–Up paths. The comparison table is shown in Table 2.

**Figure 7:**
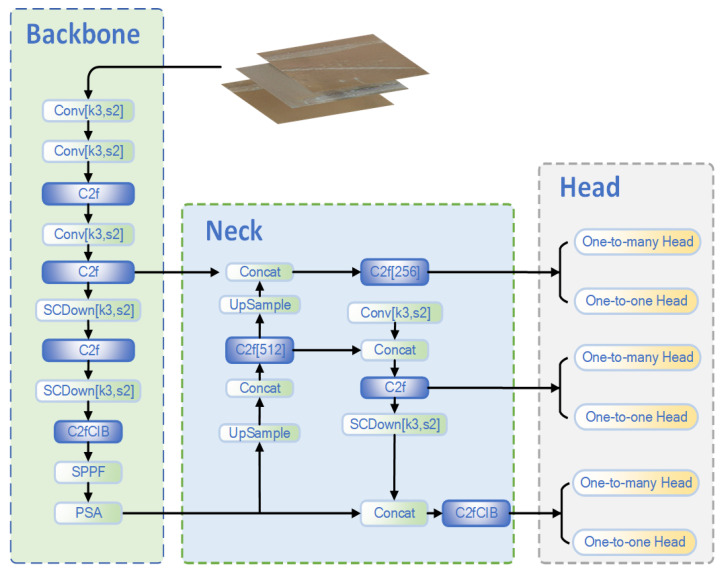
YOLOv10 network structure diagram.

Therefore, YOLOv10 is selected to complete the defect detection task. The specific structure diagram is shown in Figure 7.

YOLOv10 integrates a variety of innovative modules, enhancing feature extraction, multi-scale fusion, and detection accuracy. Its backbone network employs multiple convolutional operations (with a 3 × 3 convolution kernel and a stride of 2). Through downsampling, it reduces the resolution of the feature map, minimizing computational overhead and improving efficiency. The network incorporates an optimized C2f module. This module improves feature fluidity and reduces redundant calculations by means of cross-stage partial feature fusion. The SCDown module effectively preserves important features during downsampling, preventing information loss. The SPPF layer aggregates features through multi-scale pooling, strengthening the model’s ability to capture different receptive fields. The introduction of the PSA (Position-Sensitive Attention mechanism) further increases the focus on key areas, significantly enhancing the model’s detection capabilities in complex scenarios.

In the Neck part of YOLOv10, the detection of targets of different sizes is optimized through multi-scale feature fusion. Feature concatenation, upsampling, and downsampling operations effectively combine the high-resolution features of the shallow layers and the low-resolution features of the deep layers, constructing a semantically rich network architecture. Multi-channel C2f modules (such as 256-channel and 512-channel) further enhance the feature extraction ability, ensuring that the features of both small and large targets can be fully captured.

The detection head design of YOLOv10 supports multi-task learning and includes one-to-many and one-to-one detection heads, which are suitable for multi-class object detection and precise identification of specific targets. This architecture improves the network’s adaptability, enabling it to perform outstandingly in applications such as autonomous driving, surveillance, and robotics.

Compared with YOLOv8, YOLOv10 significantly enhances the ability to detect small targets, its performance in complex backgrounds, and its adaptability to multi-scale targets. The PSA and improved C2f modules strengthen the model’s perception ability in complex scenarios, reduce redundant calculations, and accelerate the inference speed. The SPPF and SCDown modules further improve the detection effect of multi-scale targets. Especially in scenarios involving small targets and overlapping targets, YOLOv10 demonstrates greater robustness and accuracy.

## 3. Experimental Section

### 3.1. Image Preprocessing

In this experimental section, we conducted a detailed analysis of the design, parameter selection, and training process of the Zero-DCE++ network and evaluated the experimental results.

The core architecture of Zero-DCE++ is based on depthwise separable convolution. This approach can effectively extract image features while reducing the computational load and the number of parameters. To adapt to input images of different resolutions, the network incorporates an upsampling module to adjust the size of the output image.

Zero-DCE++ introduces multiple loss functions, enabling the network to enhance the brightness of images while maintaining their naturalness and structural information. Among them, the total variation (TV) loss is used to reduce noise and keep the image smooth. The spatial loss (Lspa) and color loss (Lcolor) ensure that the enhanced image is consistent with the original image in terms of spatial structure and color distribution, respectively. The exposure loss (Lexp) helps adjust the exposure of the image to avoid it being too dark or too bright. The combination of all these loss functions allows the model to comprehensively consider the brightness, contrast, color, and structural information of the image during the optimization process, thus generating more natural and detail-rich enhanced images.

During the training process, Zero-DCE++ is optimized using the Adam optimizer. The learning rate is set to 0.0001, the weight decay is 0.0001, and the gradient clipping threshold is 0.1 to ensure the stability of training. The training batch size is set to 8, and the training lasts for 100 epochs. The model parameters are saved every 10 iterations. The weighting coefficients of the loss functions are repeatedly tuned. Eventually, manually set coefficients for each loss term are used, such as a weight of 1600 for the TV loss and a weight of 5 for the color loss.

**Figure 8:**
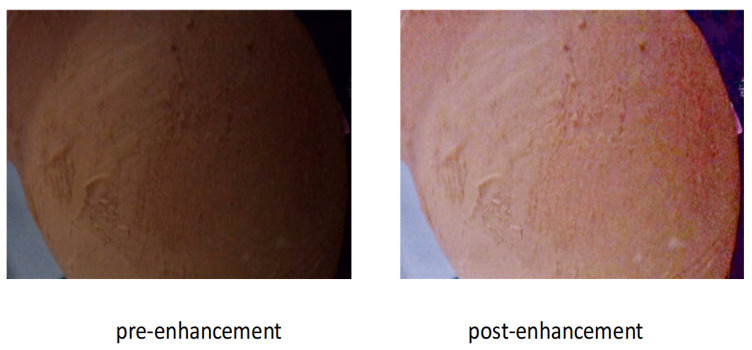
Illumination enhances contrast low-light image enhancement: original (**left**) vs. SG-LLIE result (**right**).

During the experiment, we conducted multiple tests for different scaling factors and found that when the scaling factor is set to 1, the model can achieve a good balance between computational efficiency and enhancement quality, as shown in Figure 8.

### 3.2. Image Generation

To address the challenges posed by the limited availability of cultural relic images—specifically, only 95 high-quality ceramic artifact images were collected—classical data augmentation techniques, including horizontal flipping, random rotation, scaling, and color jittering, were applied to increase the diversity of the input dataset. These methods are particularly effective in small-sample scenarios, as they preserve structural features while simulating variations in viewpoint and lighting, thereby enhancing model stability. However, the diversity of the synthesized images remained limited when using these techniques alone.

To further improve model robustness and synthesis quality, a two-stage data augmentation strategy was adopted. The dataset expanded through traditional augmentation was used to train a FastGAN model on an NVIDIA RTX 4090 GPU, with DiffAugment employed as an adaptive augmentation strategy during adversarial training. This approach continuously improved image quality while reducing the risk of overfitting. A total of 500 synthetic images were generated using the trained FastGAN model, effectively expanding the dataset while preserving the visual and structural characteristics of real samples.

The network architecture consists of a Generator and a Discriminator, both of which are implemented using convolutional and deconvolutional layers. The generator is responsible for generating fake images from random noise, while the discriminator is tasked with distinguishing between real and generated images. The parameters of the generator and the discriminator are initialized through the weights_init function. During the training process, the Adam optimizer is used with a learning rate of 0.0002 and beta1 of 0.5.

The generator and the discriminator are trained alternately. In each iteration, first, the discriminator is trained to evaluate real and fake images. After calculating the loss, backpropagation is performed to update the parameters. Then, the generator is trained by optimizing the discriminator’s output for the images it generates, with the goal of making the discriminator unable to distinguish the generated fake images.

During the training process, perceptual loss is used as a regularization term to help the generated images retain high-level features. The discriminator’s loss function combines the standard adversarial loss and the perceptual loss. The latter calculates the differences between the generated images and the real images, as well as the differences between different image reconstructions. The goal of the generator is to minimize the adversarial loss, that is, the probability that the generated images are misclassified as real images by the discriminator. This drives the generator to produce more realistic images.

The training is set to 50,000 iterations, with a batch size of nine images per iteration. The training is carried out on a single GPU, and two data loading worker threads are used to improve the efficiency of data preprocessing.

**Figure 9:**
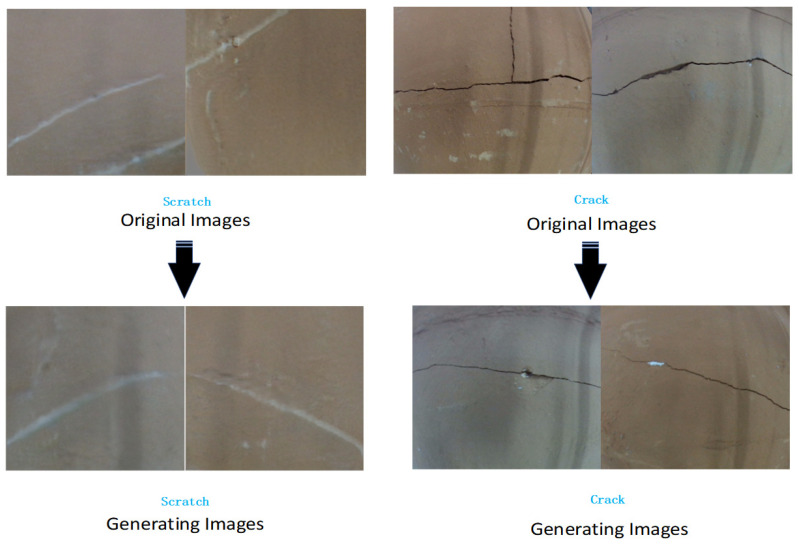
Image generation using FastGAN: real samples (**top**) vs. generated images (**bottom**).

The model and the generated images are saved every 100 iterations, and a complete checkpoint (including the weights of the generator and the discriminator and the optimizer state) is saved every 5000 iterations. The saved images can be used to visualize the training progress of the model. The output images will go through inverse normalization and be saved in the specified output directory. The use of data augmentation, multi-resolution datasets, and the perceptual loss function has improved the quality of the generated images and the robustness of the model, see Figure 9.

**Figure 10:**
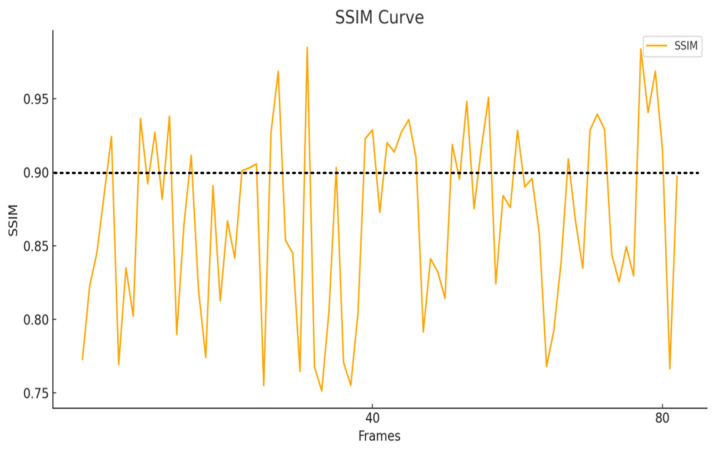
The curve of the results of the evaluation index SSIM.

**Figure 11:**
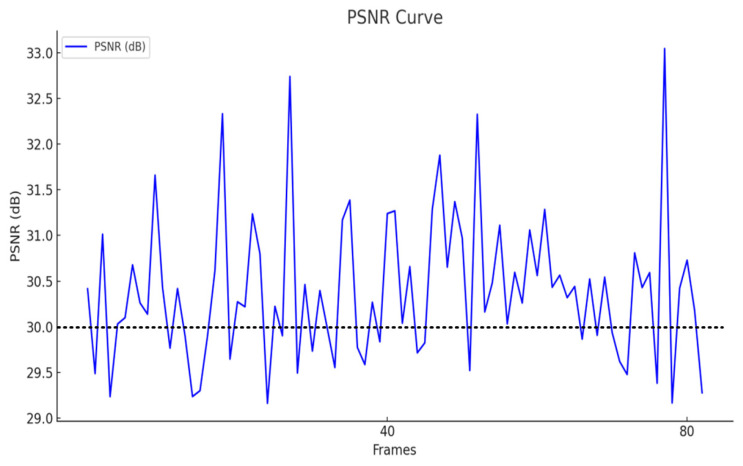
The curve of the results of the evaluation index PSNR.

Figure 10 and Figure 11 show a comparative analysis of the PSNR (Peak Signal-to-Noise Ratio) and SSIM (Structural Similarity Index) metrics between the precious pottery images generated based on FastGAN and the corresponding real images. These metrics are used to evaluate the reconstruction and structural fidelity of the generated images in the pottery defect detection task. The PSNR reflects the pixel-level reconstruction quality of the generated images. Its value ranges from 27.5 dB to 31.5 dB, with an average value of 28.89 dB. The SSIM measures the structural similarity and perceived realism of the images. Its value ranges from 0.70 to 1.00, with an average value of 0.868. The results indicate that the generation method based on FastGAN can effectively reconstruct the detailed features of precious pottery, providing reliable data support for subsequent pottery defect detection based on images. At the same time, it effectively balances pixel-level accuracy and overall visual consistency.

The average PSNR is approximately 28.89 dB, and the average SSIM is about 0.868. This indicates that the precious pottery images generated based on FastGAN exhibit high stability in terms of reconstruction quality and structural similarity. Specifically, the PSNR metric reflects the reconstruction performance of the generated images at the pixel-level accuracy. The level close to 30 dB indicates that the noise difference between the generated images and the real images is small. The SSIM value close to 0.9 indicates that the generated images have achieved a high degree of similarity in retaining structural information and visual consistency.

The performance of the above-mentioned metrics has laid a solid foundation for the task of detecting defects in precious pottery based on images. The high PSNR value ensures that minor defects can be accurately restored, while the high SSIM value helps to identify more complex structural defects. This is of great significance for the non-destructive testing of high-value items such as precious pottery.

In the image quality evaluation of pottery cultural relic defect detection, SSIM (Structural Similarity Index) and PSNR (Peak Signal-to-Noise Ratio) are adopted as the core evaluation indicators. The results show that the average value of SSIM reaches 0.868, indicating that the reconstructed images are highly consistent with the original images at the structural information level. They can accurately preserve the detailed textures and overall structural features of pottery images. At the same time, the average value of PSNR is 28.89 dB, reflecting that the error between the reconstructed images and the original images is small, and the reconstructed results exhibit high clarity in visual quality. These evaluation results indicate that the adopted method has significant performance advantages in the task of pottery cultural relic image reconstruction, providing a reliable data foundation and technical support for accurate defect detection and cultural relic protection.

### 3.3. Defect Detection Results

The dataset used in this study comprises a total of 595 images, including 95 real high-quality ceramic artifact images and 500 synthetic images generated using FastGAN. Classical augmentation techniques—such as flipping, rotation, scaling, and color jittering—were initially applied to the real images to enhance data diversity. The expanded dataset was then used to train the GAN model, which in turn provided synthetic samples with improved variability and realism.

To ensure robust and unbiased evaluation of the defect detection system, a 5-fold cross-validation strategy was adopted. The entire dataset was randomly partitioned into five equally sized folds. In each iteration, four folds (80%) were used for training and one fold (20%) for validation. This approach ensured that each image was used for both training and validation exactly once across the five iterations. To mitigate overfitting, training included early stopping based on validation loss, L2 weight decay, and dropout layers in the detection head.

**Figure 12:**
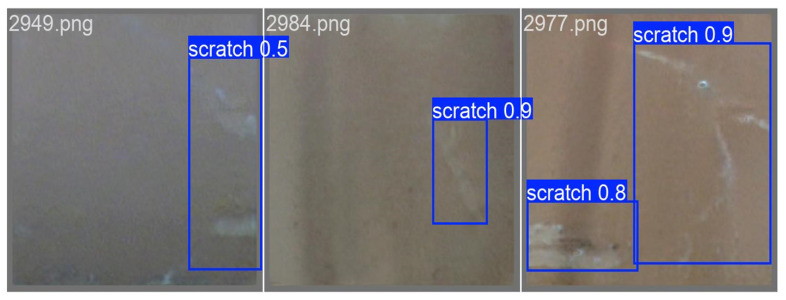
The result of scratch defect detection.

**Figure 13:**
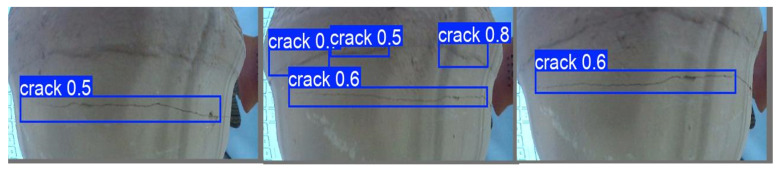
The result of crack defect detection.

The collected ceramic defect images were subjected to a comprehensive detection process, as depicted in Figure 12 and Figure 13. Notably, the defects present on the ceramics were precisely marked with blue frames for clear visualization and subsequent analysis. In the context of this research, the identified defects were categorized and named as “crack” and “scratch”, respectively. The numerical values appended to these defect labels signify the probabilities of their accurate identification as actual defects, providing a quantitative measure of the detection reliability.

The detection results demonstrated a remarkably high level of accuracy. Specifically, as illustrated in the subsequent figure, a random selection of six inner-sleeve magnetic images was analyzed. These images collectively contained a total of 10 defects. Intriguingly, the detection algorithm successfully and completely identified all the ceramic defects within these six images, achieving a total of 10 correct detections. This outcome not only validates the efficacy of the employed detection methodology but also highlights its potential for practical applications in the quality control and inspection of ceramic products.

## 4. Discussion

### 4.1. Defect Detection Evaluation

In this paper, based on YOLOv10, the experimental environment uses the Python v.3.10.12. language, and the GPU is an Nvidia RTX 4090 graphics card. Defect-generated ceramic images are used for defect detection, and the following evaluation metrics are adopted to conduct a quantitative analysis of the training results of YOLOv10.

The performance of the model can be comprehensively evaluated through multiple indicators. Among them, precision, recall, average precision (AP), and mean average precision (mAP) are commonly used evaluation criteria.

Precision measures the proportion of actual positive samples among all samples predicted as positive by the model. The formula is:

(3) $$Precision=\frac{TP}{TP+FP}$$

Here, (True Positives) represents the number of samples that the model correctly predicts as positive, and (False Positives) represents the number of samples that the model incorrectly predicts as positive. Precision reflects the accuracy of the model when predicting positive samples. High precision means that most of the samples predicted as positive by the model are actually positive samples.

Recall, on the other hand, measures the proportion of samples that are correctly predicted as positive by the model among all actual positive samples. The formula is:

(4) $$Recall=\frac{TP}{TP+FN}$$

Here, FN (False Negatives) represents the number of samples that the model incorrectly predicts as negative. A high recall rate means that the model can identify more positive samples, and the false negative rate is low. To comprehensively consider precision and recall, the average precision (AP) is usually calculated. This metric reflects the overall performance of the model under different recall thresholds. AP is the weighted average of precision and recall under different thresholds, so it can comprehensively evaluate the performance of the model in different prediction scenarios.

In a multi-class detection task, the mean average precision (mAP) is used as a measure of overall performance and is usually the average of AP values of various classes. The mAP value reflects the detection ability of the model in each category and can provide a quantitative evaluation of the model’s multi-class detection performance. The formula is:

(5) $$AP=\int_{0}^{1}Precision(Recall)d(Recall)$$

(6) $$mAP=\frac{1}{N}\sum_{i=1}^{N}AP_{i}$$

Here, C is the total number of categories, and APi is the average precision of the i-th category.

To further verify the performance of the model, different Intersection over Union (IoU) thresholds are usually set to evaluate the model’s performance under different overlap requirements. The change of IoU will affect the calculation results of precision, recall, AP, and mAP.

These metrics are of great significance in the object detection task. Through them, we can comprehensively analyze the detection ability of the model, especially its performance in balancing precision and recall, and then optimize the model design and application.

For the two defect types, crack and scratch, the specific parameters of YOLOv8 and YOLOv10 are shown in Table 3.

Based on the analysis of the data in Table 2, YOLOv10 outperforms YOLOv8 in the ceramic defect detection task. Specifically, the Recall values of YOLOv10 are 85.08% (Crack) and 85.41% (scratch), which are higher than 83.78% (Crack) and 81.73% (scratch) of YOLOv8. This indicates that the model has a more comprehensive coverage of defect samples. Secondly, the Precision values of YOLOv10 are 90.24% (Crack) and 93.04% (scratch), which are significantly higher than 87.91% (Crack) and 88.29% (scratch) of YOLOv8. This shows that it has a stronger ability to reduce false alarms. Finally, the mAP values of YOLOv10 reach 91.71% and 92.04%, respectively, both exceeding 90%, which are higher than 88.48% (Crack) and 89.71% (scratch) of YOLOv8. This reflects the superiority and stability of the model’s overall detection performance.

As shown in Table 4, the proposed defect detection system demonstrates consistent performance across all five folds of the cross-validation. The mean Average Precision (mAP) across the folds is 0.831, with a low standard deviation of ±0.006, indicating stable detection accuracy. Similarly, the average Precision and Recall are 0.844 and 0.765, respectively, both with minimal variance across folds. These results suggest that the model effectively generalizes to unseen data and is not prone to overfitting, despite being trained on a relatively limited dataset.

The consistent performance is attributed to the combination of classical augmentation, GAN-based synthesis, and a balanced data splitting strategy. The use of 5-fold cross-validation further strengthens the reliability of the evaluation by ensuring that all images are validated at least once across different configurations. This supports the robustness of the proposed detection pipeline and confirms its applicability in real-world scenarios.

**Figure 14:**
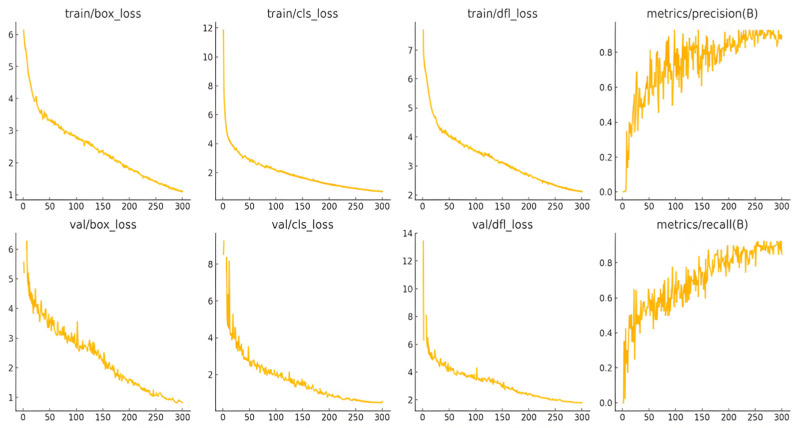
YOLOv10 training process curve.

The training process of YOLOv10 is shown in Figure 14, which presents the changes of key indicators during the training and validation of the model. The loss curves of both the training set and the validation set show a downward trend, indicating that the model is gradually learning and optimizing. The training loss decreases relatively fast, while the validation loss fluctuates slightly, and the risk of overfitting may need to be paid attention to. The precision and recall curves (metrics/precision, metrics/recall) gradually rise and tend to be stable, reflecting the improvement of the model’s prediction ability. Overall, the training effect of the model is good.

As illustrated in Table 5, the YOLOv10-based detection framework demonstrates superior overall performance compared to other state-of-the-art baseline models. It achieves the highest mean Average Precision (mAP) of 0.831, indicating improved localization and classification capabilities for multiple defect categories. Its Precision value of 0.844 reflects a lower false-positive rate and enhanced reliability in correctly identifying defect instances, while its Recall of 0.765 suggests that fewer true defects are missed during detection.

In contrast, YOLO-pdd achieves an mAP of 0.816, a Precision of 0.830, and a Recall of 0.754, showing competitive results but slightly lower detection robustness than YOLOv10. BL-YOLOv8 records an mAP of 0.809, with Precision and Recall values of 0.828 and 0.748, respectively, indicating a minor degradation in both localization accuracy and defect coverage compared to YOLOv10. YOLOv5 exhibits the lowest performance among the YOLO-based models, with an mAP of 0.804, a Precision of 0.822, and a Recall of 0.742, demonstrating weaker adaptability to small-scale or low-contrast defects in complex wall textures. Traditional deep learning architectures such as UNet variants and ResNet-CNN show noticeably lower performance, particularly in Recall (0.724 and 0.701, respectively), highlighting their limited generalization capabilities in this task.

### 4.2. Ablation Study

Table 6 summarizes the performance of three different image generation methods—FastGAN, StyleGAN2, and Diffusion Model—used for data augmentation in this study. The evaluation metrics include Peak Signal-to-Noise Ratio (PSNR), Structural Similarity Index Measure (SSIM), and total training time in hours.

The Diffusion Model achieves the highest image quality, with a PSNR of 35.01 dB and an SSIM of 0.932, indicating superior fidelity and structural preservation. StyleGAN2 also delivers high performance (PSNR: 33.72 dB; SSIM: 0.913), albeit with moderate computational cost. FastGAN, while achieving slightly lower quality metrics (PSNR: 30.89 dB; SSIM: 0.903), significantly outperforms the others in training efficiency, completing the process in just 2.8 h.

These results reveal a clear trade-off between quality and efficiency. While Diffusion-based models generate the most visually consistent outputs, their high computational demands may not be practical in resource-constrained or real-time scenarios. FastGAN, on the other hand, offers a more balanced solution, delivering acceptable image fidelity with minimal training overhead, making it particularly well-suited for rapid data augmentation in small-sample cultural heritage applications.

To investigate the impact of different data augmentation strategies on detection performance, we conducted a series of ablation experiments using YOLOv8 and YOLOv10 as detection backbones. The tested augmentation strategies include traditional image transformations, synthetic sample generation using FastGAN, and illumination correction via Zero-DCE++. The results are reported in terms of mean Average Precision (mAP), precision, and recall.

As shown in Table 7, YOLOv10 consistently outperforms YOLOv8 across all augmentation configurations. In the baseline scenario (Experiment A), where only FastGAN is used without traditional augmentation or illumination enhancement, YOLOv10 achieves an mAP of 89.93%, a precision of 89.51%, and a recall of 84.02%, which already surpasses the traditional-only setup (Experiment B) that yields 84.62% mAP. This indicates that GAN-based augmentation provides more diverse and informative synthetic data than classical geometric transformations alone.

When illumination enhancement is introduced via Zero-DCE++ in addition to FastGAN (Experiment C), further improvements are observed, particularly in recall (YOLOv10: 85.08%). Combining traditional augmentation with FastGAN (Experiment D) leads to a strong synergy, increasing YOLOv10′s mAP to 90.04%. The highest performance is achieved in Experiment E, which integrates all three augmentation strategies. In this configuration, YOLOv10 reaches an mAP of 91.88%, a precision of 91.64%, and a recall of 85.25%.

These results suggest that combining spatial, generative, and illumination-based augmentations yields the most robust training set, particularly for small-sample, high-variance domains such as cultural heritage artifact analysis. YOLOv10 benefits more significantly from these enhancements than YOLOv8, owing to its stronger backbone and improved detection head, making it a preferred choice for high-precision applications.

### 4.3. Generalization Performance on Multi-Material Cultural Artifacts

**Figure 15:**
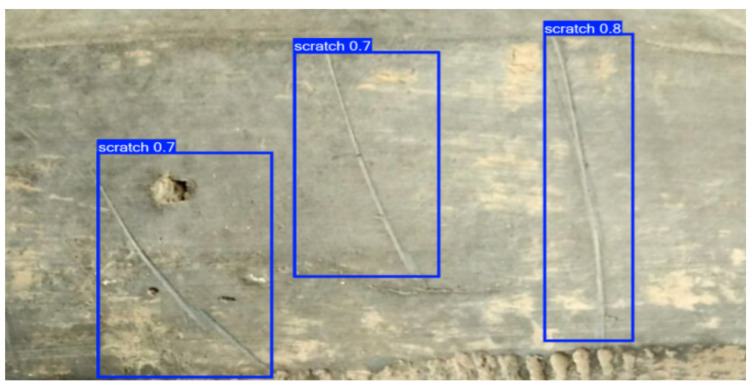
Scratch defect detection results of actual unearthed cultural relics.

**Figure 16:**
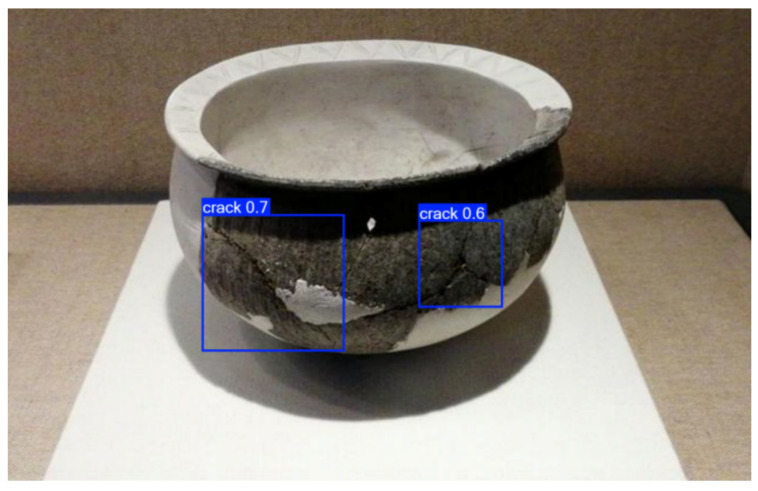
Crack defect detection results of actual unearthed cultural relics.

In the field of cultural heritage research and conservation, to further explore the transferability of training models, we applied a small-sample-based defect visual detection method to archaeological artifacts made from different materials. Figure 15 shows the surface condition of a Yangshao culture pottery piece, which dates back to approximately 7000 to 5000 years ago and is an important Neolithic culture in the middle reaches of the Yellow River. The image highlights a “scratch 0.8” mark, likely caused by friction or external forces during the pottery’s use, reflecting the daily life and usage habits of the time. Figure 16 displays pottery from the Shanshan and Kuahuqiao cultures, with the Shanshan culture dating back to approximately 11,000 to 8500 years ago and being one of the origins of rice farming. The surface of the pottery in the image shows visible damage and cracks, which are common defects in excavated ceramics, reflecting the firing techniques and usage methods of the time. The degree and location of the damage, as well as the form and direction of the cracks, may be related to the cooling process after firing and the changes in the burial environment, offering valuable insights into the social life, economic patterns, and cultural continuity of these two cultural periods.

The detection method we used can accurately identify defects in pottery from different cultural backgrounds. In the experiment, the scratches on the Yangshao culture pottery and the cracks on the Shanshan and Kuahuqiao culture pottery were precisely marked. The method offers several notable advantages: first, it requires few samples, making it ideal for precious and rare archaeological artifacts; second, it achieves high detection accuracy, capturing defects of various types; and third, it is highly adaptable, capable of effectively detecting pottery of different materials and preservation conditions. These features make the method highly promising for applications in cultural heritage conservation, providing reliable technical support for the preservation and study of excavated pottery.

**Figure 17:**
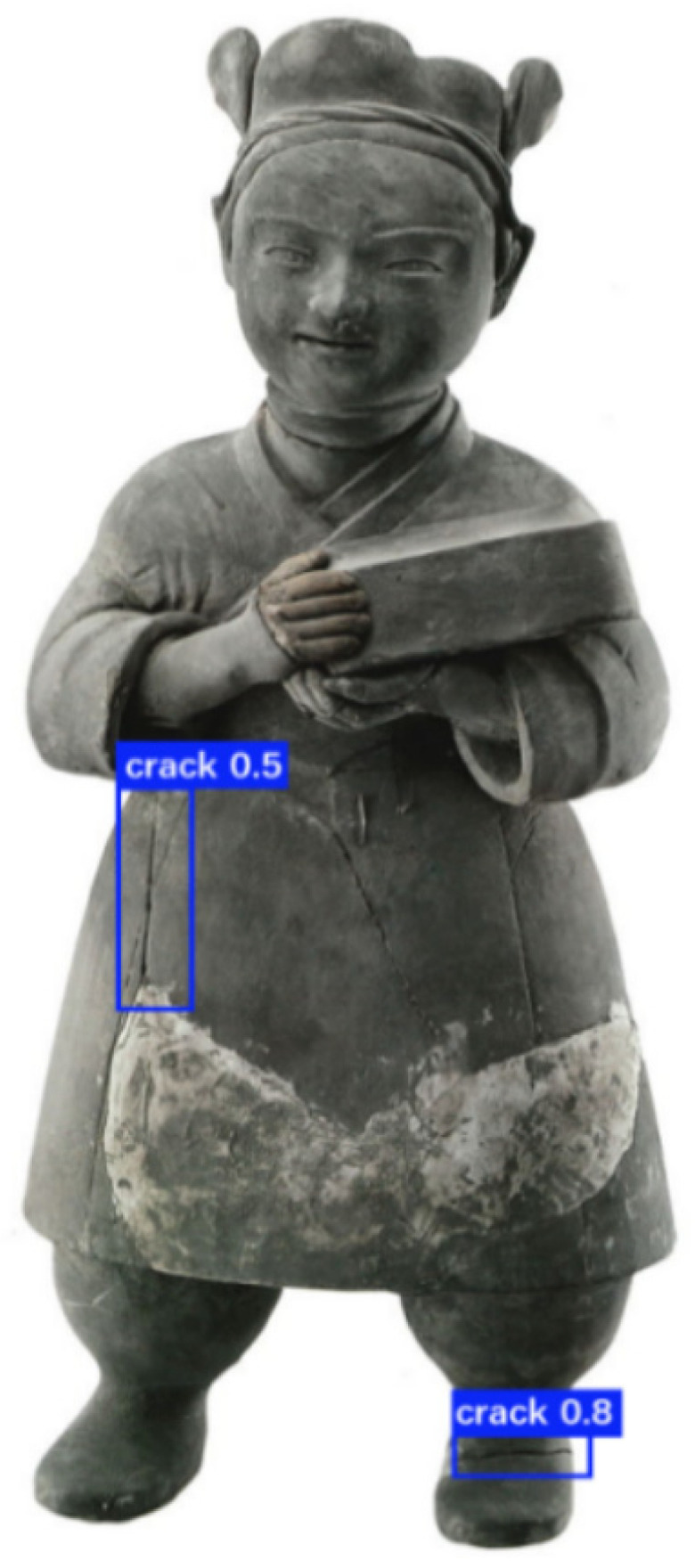
Defect detection results of stone statues.

**Figure 18:**
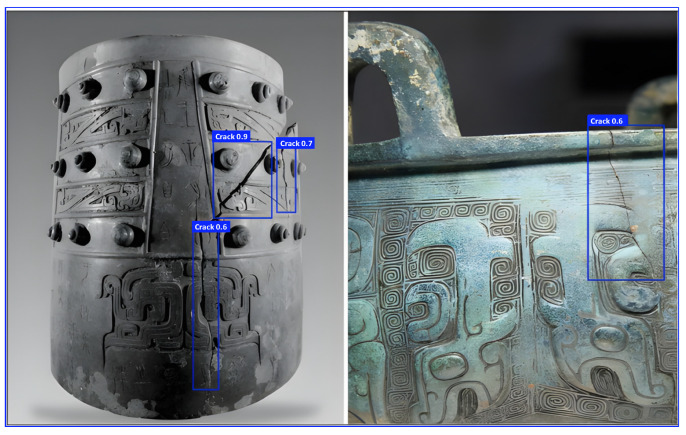
Defect detection results of bronzer.

Through the detection of defects in excavated pottery images, our method has proven its significant application value and broad development prospects in the field of cultural heritage conservation. To further explore the transferability of the trained model, we applied this method to detect defects in artifacts made from different materials. Figure 17 presents a stone figurine from the Warring States period, which, with its unique shape and meticulous craftsmanship, showcases the social customs and aesthetic tastes of that era. Two cracks were detected on the stone figurine, with confidence scores above 0.5. Figure 18 shows a bronze ding and chime bells from the Spring and Autumn and Warring States periods. The bronze ding is adorned with exquisite patterns and exudes grandeur, while the chime bells are neatly arranged, once resonating with the elegant melodies of history. We successfully detected four defects in the image, with all cracks having confidence scores above 0.6.

The detection method we used demonstrated outstanding performance and significant advantages when applied to artifacts from different materials and time periods. The main features of this method include high precision, strong transferability, and high efficiency. First, it can accurately identify even the smallest defects, such as the cracks on the stone figurine and the bronze ding, with precise confidence scores providing data support for artifact restoration and research. Second, the method has strong transferability and can effectively detect defects in artifacts made from various materials, whether they are hard stone or unique bronze, ensuring accurate detection. Finally, the method requires no complex preprocessing, does not damage the artifacts, and delivers results quickly, greatly improving detection efficiency, especially for precious and limited artifacts.

While promising visual results for stone and bronze defect detection are presented in Figure 17 and Figure 18, these were obtained using the original ceramic-trained model without any fine-tuning. This zero-shot transfer indicates the model’s potential to generalize low-level features such as cracks and abrasions across different material domains. However, it is important to note that material properties—such as surface reflectivity, texture granularity, and inherent noise—differ significantly across ceramic, stone, and bronze artifacts. These differences may affect detection accuracy and limit the model’s robustness when applied beyond its training domain. Furthermore, the current dataset suffers from constraints in both scale and diversity, consisting of only 95 real ceramic images and 500 synthetic samples generated via FastGAN. While this synthetic augmentation strategy mitigates the impact of limited real data, it does not capture the full spectrum of variations found in real-world cultural heritage defects, nor does it include annotated examples from non-ceramic materials. To overcome these limitations, future work will focus on (1) collaborating with museums and cultural heritage institutions to collect real defect data from stone, bronze, and other materials; (2) constructing a multi-material benchmark dataset covering common defect types such as cracks, corrosion, and surface erosion; and (3) releasing a curated public subset of ceramic defect images with detailed annotations for academic research. These efforts will enable more rigorous cross-domain evaluations, support transfer learning, and promote reproducibility and open collaboration in the field of heritage conservation.

## 5. Conclusions

In the realm of precious ceramic defect detection, the scarcity of training sets poses significant challenges. To tackle these issues head-on, this paper presents a comprehensive approach that integrates virtual image generation based on FastGAN and defect detection using YOLOv10. The following are the key contributions of this research:

1. Develop a novel and high-efficiency defect detection method specifically for unearthed pottery, which uses advanced machine vision technology to accurately and quickly identify defects with an accuracy of over 90%.

This paper introduces a novel defect detection method for unearthed pottery, leveraging the power of machine vision technology. Historically, the identification of defects in pottery cultural relics has been a labor-intensive and time-consuming process, often resulting in low efficiency. However, the proposed method revolutionizes this scenario. By harnessing advanced algorithms and state-of-the-art techniques, it enables highly efficient and precise detection of pottery defects. Rigorous experiments and evaluations have demonstrated that this method achieves an astonishing accuracy rate of over 90%, marking a significant leap forward in the field of pottery defect detection.

2. Introducing an Innovative Image Data Augmentation Algorithm

In order to solve the problem of difficult image acquisition of pottery relics, this paper uses an innovative use of an image data enhancement algorithm, which effectively expands the available data set, and shows remarkable stability in reconstruction quality and structural similarity. The average PSNR value is 28.89 dB, the SSIM value is close to 0.9, and the average PSNR value is 28.89 dB. More rich and diverse image data are provided for model training. In this way, this paper successfully solves the challenge caused by the limited amount of data and significantly improves the robustness and accuracy of the cultural relic recognition model. This not only improves the performance of the defect detection system but also provides a new methodology and solution for the research of image-based cultural heritage protection, and injects new impetus into the cause of cultural heritage protection.

3. Demonstrating Wide-Ranging Applicability Across Different Cultural Relic Materials

The significance of this research extends far beyond pottery cultural relics. The proposed method has shown remarkable adaptability when applied to cultural relics made of other materials. Through meticulous fine-tuning and optimization of the model, it has the potential to be customized for a wide variety of cultural relics, enabling defect detection across different materials such as stone, bronze, and more. This broad applicability not only showcases the versatility of the method but also holds great promise for the future of cultural relic protection. It offers a unified approach that can be tailored to meet the specific needs of different types of cultural relics, providing a valuable tool for conservators, archaeologists, and researchers in the field. By facilitating the early detection and analysis of defects, this method can contribute to the long-term preservation and conservation of our precious cultural heritage, ensuring that these invaluable artifacts are safeguarded for future generations.

In summary, this research makes significant contributions to the field of precious ceramic defect detection. By integrating FastGAN-based virtual image generation and YOLOv10 for defect detection, it effectively addresses the challenges brought about by the scarcity of training sets. The proposed high-efficiency defect detection method for unearthed pottery revolutionizes traditional detection processes with its high accuracy. The innovative image data augmentation algorithm successfully overcomes the data shortage problem, enhancing the model’s performance and offering new solutions for image-based cultural heritage protection. Moreover, the wide-ranging applicability of the method across different cultural relic materials broadens its utility, providing a unified approach for various cultural relics. Overall, this research not only enriches the academic research in the field of cultural relic protection but also has practical implications for the long-term preservation of precious cultural heritage.

Although this study focuses on detecting surface defects of ceramic products, future work will explore extending this framework to texture classification and style analysis. Compared with defect detection, texture recognition pays more attention to the consistency of the overall surface pattern and style, which requires corresponding adjustments in the network architecture and data processing. The attention mechanism and pyramid structure can further enhance the network’s ability to model texture granularity and changes. The loss function will shift from focusing on localization to classification-based goals, such as cross-entropy or contrastive loss. Additionally, texture datasets usually require image-level annotations and may benefit from block-based sampling strategies to capture high-resolution details. These improvements will enable the proposed system to support applications in broader fields such as ceramic process classification, repair planning, and artifact provenance analysis.

## Figures and Tables

**Table 1:** Comparison of FastGAN, StyleGAN2, and diffusion models across key criteria.

Criteria	FastGAN	StyleGAN2	Diffusion/Stable Diffusion
Training Speed	Fast; can be trained within hours	Very slow; requires extensive training time	Very slow; training typically takes several days
Data Requirements	Performs well with limited data	Requires a large amount of high-quality data	Requires moderate to large-scale datasets
Output Quality and Visual Details	Moderate visual quality; sufficient for use	Very high-quality image generation	Excellent detail and visual fidelity
Adaptability to Niche Styles	High adaptability to rare or historical styles	Limited flexibility for uncommon styles	Requires additional effort to adapt to niche aesthetics
Suitability for Rapid Prototyping	Highly suitable	Not suitable	Not suitable
Hardware Requirements	Runs efficiently on mid-range GPUs	Requires high-end, often multi-GPU setups	Requires powerful GPUs and large memory capacity

**Table 2:** Feature comparison between YOLOv8 and YOLOv10.

Characteristic	YOLOv8	YOLOv10
Architecture	Improved based on YOLOv5, with enhanced feature extraction network and attention mechanisms.	Further optimization of YOLOv8, incorporating Transformer and Graph Neural Networks (GNN).
Key Innovations	Enhanced small object detection, introduced attention mechanisms, and optimized computational graph.	Introduction of Transformer and GNN to enhance global feature modeling, further improving detection accuracy.
Computational Efficiency	Optimized convolution operations, improving computational speed and adapting to embedded devices.	Support for multi-task learning, optimized computational graph, suitable for edge computing and real-time tasks.
Detection Accuracy	Improved robustness for small objects and complex scenes.	Further improved accuracy, especially in dynamic backgrounds and complex scenes.
Model Size	Optimized for smaller models, suitable for embedded and resource-constrained devices.	Further lightweight optimization, maintaining high accuracy while improving efficiency.
Inference Speed	Fast inference speed, suitable for real-time applications.	Even higher inference speed, suitable for high-dynamic real-time environments.
Integration of New Technologies	Attention mechanisms (SE blocks, ECA, etc.) enhance feature extraction capabilities.	Integration of Transformer and Graph Neural Networks (GNN), enhancing cross-domain detection capabilities.

**Table 3:** Training parameters for ceramic defects.

	Recall		Precision		mAP	
	YOLOv8	YOLOv10	YOLOv8	YOLOv10	YOLOv8	YOLOv10
Crack	83.78%	85.08%	87.91%	90.24%	88.48%	91.71%
Scratch	81.73%	85.41%	88.29%	93.04%	89.71%	92.04%

**Table 4:** Cross-validation results of the proposed detection model (5-fold).

Fold	mAP	Precision	Recall
1	0.829	0.841	0.762
2	0.833	0.846	0.766
3	0.826	0.843	0.760
4	0.837	0.847	0.769
5	0.831	0.844	0.767
Avg.	0.831	0.844	0.765
Std.	±0.006	±0.002	±0.003

**Table 5:** Comparison of detection performance among different methods.

Method	mAP	Precision	Recall
YOLOv10	0.831	0.844	0.765
ResNet-CNN	0.794	0.823	0.701
UNet variant	0.812	0.835	0.724
YOLO-pdd	0.816	0.830	0.754
BL-YOLOv8	0.809	0.828	0.748
YOLOv5	0.804	0.822	0.742

**Table 6:** Comparison of image generation methods for data augmentation.

Augmentation Method	PSNR (dB)	SSIM	Training Time (Hours)
FastGAN	30.89	0.903	2.8
StyleGAN2	33.72	0.913	10.3
Diffusion Model	35.01	0.932	16.9

**Table 7:** Comparison of detection performance under different augmentation strategies on YOLOv8 and YOLOv10.

Number	Traditional Data Augmentation	FastGAN	Zero-DCE++	YOLOv8			YOLOv10		
				mAP (%)	Precision (%)	Recall (%)	mAP (%)	Precision (%)	Recall (%)
A				87.62	88.29	81.73	89.93	89.51	84.02
B	√			82.15	84.57	78.62	84.62	87.91	83.78
C		√		88.48	89.73	83.91	89.71	90.24	85.08
D	√	√		89.10	88.10	82.76	90.04	89.24	85.08
E	√	√	√	89.27	90.38	83.59	91.88	91.64	85.25

## Data Availability

The original contributions presented in this study are included in the article. Further inquiries can be directed to the corresponding authors.
